# A case of IgG4-related disease misdiagnosed as perirenal abscess: Case report and literature review

**DOI:** 10.1097/MD.0000000000045898

**Published:** 2025-11-14

**Authors:** Zhongze Zhou, Fengxiang Gan, Kun Zhao, Shuai Liu, Xi Xiao, Wenxuan Li, Xinling Han, Wenyun Wang, Zhilong Dong

**Affiliations:** a Department of Urology, Second Hospital of Lanzhou University, Lanzhou, Gansu, China; b The Second Hospital & Clinical Medical School, Lanzhou University, Lanzhou, Gansu, China; c Tianzhu Tibetan Autonomous County People’s Hospital, Tianzhu, Gansu, China.

**Keywords:** autoimmune disease, case report, IgG4-related disease, misdiagnosis, perirenal mass, renal involvement

## Abstract

**Rationale:**

Immunoglobulin G4–related disease (IgG4-RD) is a chronic immune-mediated fibroinflammatory condition that can mimic infection or malignancy. Renal involvement may present as a perirenal mass suggestive of an abscess. This case highlights a diagnostic pitfall and a favorable response to therapy.

**Patient concerns:**

A 48-year-old man had a persistent fever for over 20 days. Imaging revealed a left renal mass. Empirical antibiotics were initiated without improvement, and he was transferred for further evaluation.

**Diagnoses:**

Laboratory tests showed elevated inflammatory markers, including C-reactive protein and interleukin-6, positive proteinase 3 antineutrophil cytoplasmic antibody, and markedly increased serum immunoglobulin G4 (IgG4). Computed tomography and magnetic resonance imaging demonstrated renal and pulmonary abnormalities. Renal biopsy confirmed dense infiltration of IgG4-positive plasma cells. The final diagnosis was IgG4-RD involving the kidneys and lungs, initially misdiagnosed as a perirenal abscess.

**Interventions:**

Systemic corticosteroid therapy was started.

**Outcomes:**

The patient experienced rapid resolution of fever and clinical improvement, with reductions in serum IgG4 and interleukin-6 levels.

**Lessons:**

IgG4-RD should be considered in patients with unexplained fever and space-occupying renal lesions unresponsive to antibiotics. Timely recognition and immunosuppressive therapy can lead to favorable outcomes and help prevent irreversible organ damage.

## 1. Introduction

IgG4-related disease (IgG4-RD) is an emerging immune-mediated systemic disorder, which has undergone significant clinical recognition since the characterization of pancreatic lesions in 2003.^[1]^ The disease is characterized by multi-organ fibrotic inflammation, elevated serum IgG4 levels, and extensive infiltration of IgG4-positive plasma cells in tissue pathology.^[2]^ The pancreas is the most commonly affected organ; however, IgG4-RD can involve any part of the body, including the salivary glands, kidneys, aorta, lungs, and retroperitoneum.^[3,4]^ The clinical manifestations often overlap with those of tumors, infectious diseases, or other immune disorders, leading to a misdiagnosis rate of up to 30% to 50%.

The clinical presentation is typically subacute, with symptoms primarily related to organ dysfunction. A history of rhinitis, asthma, nasal polyps, and sinusitis is commonly observed.^[5,6]^

In recent years, with the release of the 2019 international classification criteria, the clinical diagnostic rate has improved; however, the diagnosis and treatment of atypical cases remain challenging.^[7]^ This article reports a case of IgG4-RD with unexplained fever and retroperitoneal fibrosis as the initial presenting features. The case also demonstrated elevated serum IgG4 levels and atypical imaging findings, with the final diagnosis confirmed through multidisciplinary collaboration. This case highlights the recognition strategy for seronegative cases in clinical practice and underscores the pivotal role of histopathology in the diagnosis.

## 2. Case presentation

### 2.1. Chief complaint

The patient was admitted with a chief complaint of persistent fever lasting for over 20 days.

### 2.2. Present illness

The patient is a 48-year-old male who developed a persistent high fever over 20 days ago without any obvious precipitating factors. He sought medical attention at a local hospital, where relevant tests were performed, and ultrasound indicated an inflammatory lesion around the left kidney. Empirical antibiotic therapy was initiated, but the clinical response was unsatisfactory. Afterward, the patient and his family decided to seek further examination and treatment at our hospital. Upon detailed history taking in the outpatient department, the patient was admitted to the urology department with a diagnosis of “perirenal abscess.” Throughout the course of the illness, the patient appeared alert and in good spirits, with normal appetite, sleep, and bowel movements. There was no significant change in weight.

### 2.3. Past medical history

The patient has a history of diabetes mellitus.

### 2.4. Personal and family history

Denies any history of kidney disease.

### 2.5. Physical examination

No significant abnormalities were noted.

## 3. Auxiliary Examinations

Upon admission, the patient underwent further imaging and laboratory tests.

### 3.1. Ultrasonography

**Figure 1.**
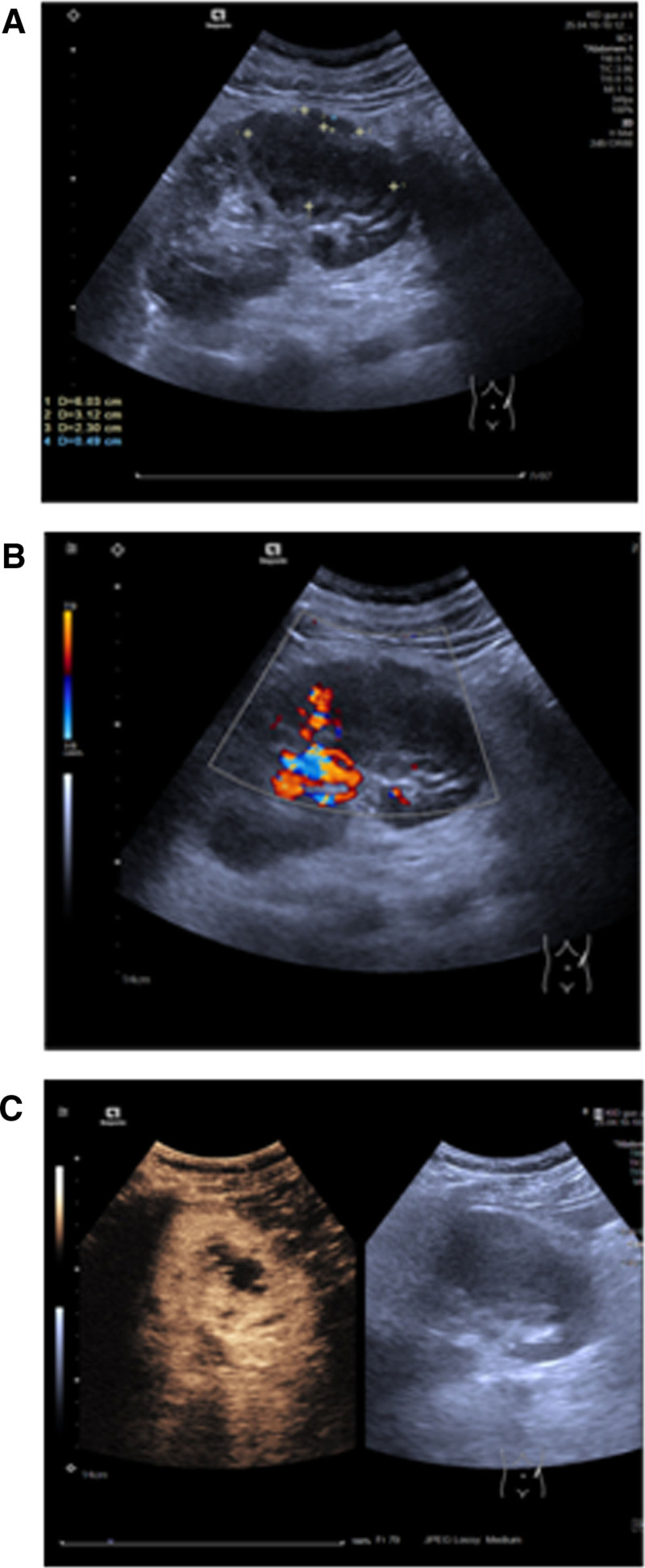
Ultrasound images (A–C). (A) The asterisk (*) demarcates a poorly defined, hypoechoic mass located at the lower pole of the left kidney. The mass demonstrates posterior acoustic enhancement, a feature that can be seen in both fluid-filled and solid inflammatory lesions. (B) The focal area of color signal (within the measurement calipers) demonstrates internal vascularity within the perirenal mass. The presence of intralesional blood flow is an atypical finding for a simple abscess, which is typically avascular, and raised suspicion for an inflammatory or neoplastic process. (C) Contrast-enhanced ultrasound (CEUS) in the early phase demonstrates moderate, heterogeneous enhancement of the peripheral rim (arrowheads) with a predominantly hypoenhanced central area. This “rind-and-core” pattern is atypical for a classic abscess and is suggestive of an inflammatory or infiltrative process such as IgG4-RD. IgG4-RD = immunoglobulin G4-related disease.

Grayscale ultrasound identified a poorly defined, hypoechoic mass at the lower pole of the left kidney, demonstrating posterior acoustic enhancement (Fig. 1A). Color Doppler ultrasonography revealed the presence of internal vascularity within the mass (Fig. 1B). Subsequent contrast-enhanced ultrasound performed during the early phase demonstrated moderate, heterogeneous enhancement of the peripheral rim with a predominantly hypoenhanced central area (Fig. 1C).

### 3.2. Computed tomography (CT) scan

**Figure 2.**
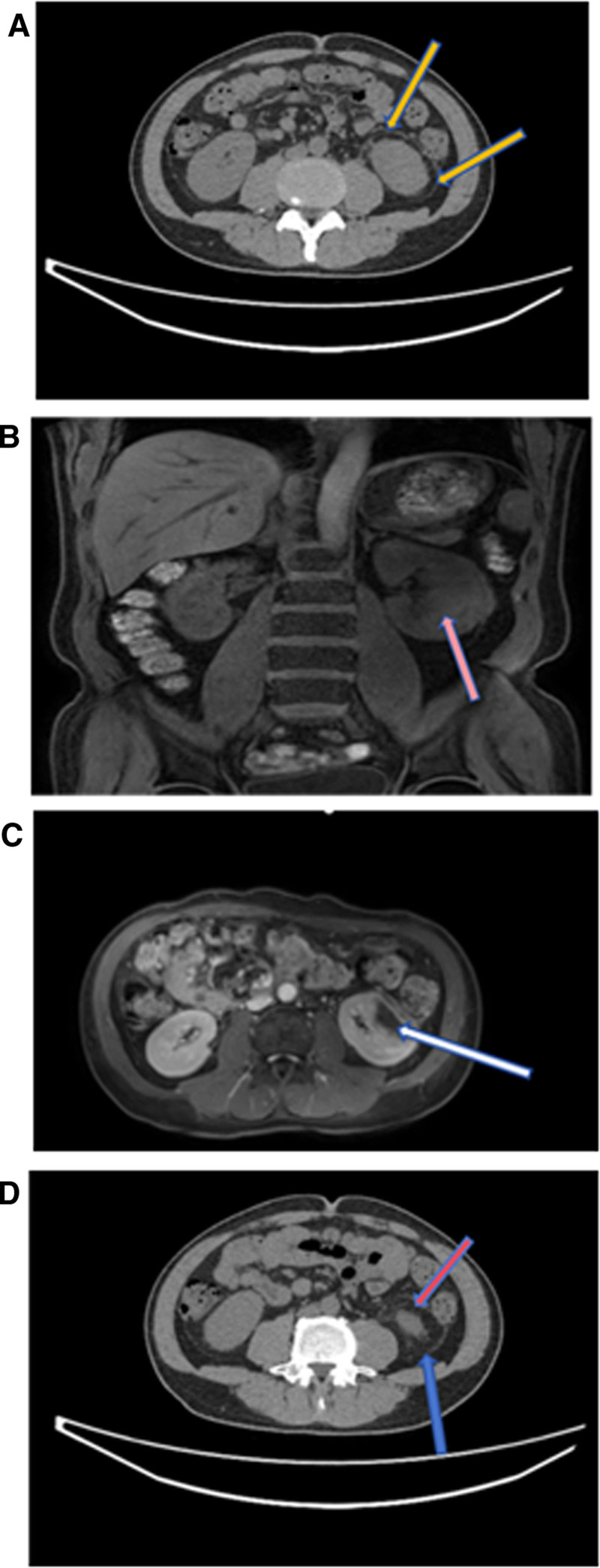
CT and MRI images (A–D). (A) The arrow points to an ill-defined, soft-tissue attenuation mass enveloping the left kidney (LK). Note the absence of a fluid-density center or rim enhancement, which are typical of an abscess. Instead, the infiltrative nature of the lesion is more consistent with retroperitoneal fibrosis or inflammatory infiltration. (B) Arrowheads highlight patchy areas of high T2 signal intensity within the left renal parenchyma and the perirenal mass. These represent sites of active inflammation or edema, which are characteristic of IgG4-RD. The black arrow indicates subtle stranding in the perirenal fat. (C) The arrow points to the extent of the infiltration process, showing the intermediate signal intensity on T1 weighted imaging. The lesion showed a mass effect, resulting in mild distortion of the renal contour. (D) Diffuse thickening and infiltration of the left perirenal fascia and Gerota fascia. Surrounded by fibroinflammatory tissue. This manifestation is a hallmark of IgG4 related retroperitoneal fibrosis. CT = computed tomography, IgG4-RD = immunoglobulin G4-related disease, IL = interleukin, MRI = magnetic resonance imaging.

A mass lesion with varying density was observed in the middle and lower poles of the left kidney (Fig. 2A), further evaluated with laboratory tests and enhanced CT imaging.

- Bilateral pulmonary interstitial changes with multiple calcified foci and small nodules were noted. These findings warrant clinical correlation to exclude alternative diagnoses.

### 3.3. Magnetic resonance imaging (MRI)

Abnormal patchy signals were seen in the lower pole of the left kidney, suggestive of a non-tumorous lesion, with infection being a likely consideration (Fig. 2B–D).

### 3.4. Laboratory tests

- White blood cell count: 16.67 × 10⁹/L (↑)
- Neutrophil percentage: 89.7% (↑)
- C-reactive protein (CRP): 268.97 mg/L (↑)
- Serum IgG: 1760.98 mg/dL (↑)
- IgM rheumatoid factor: 217.60 IU/mL (↑)
- Complement C3 and C4 levels: within normal range
- Blood glucose metabolism abnormalities with HbA1c: 6.8%
- Blood culture and urine culture: negative

The key imaging findings are presented in Figure 1 and Figure 2

### 3.5. Diagnostic process

During hospitalization, the patient continued to experience persistent high fever, despite treatment with Tylenol for infection, with the temperature fluctuating around 37.5°C. Symptomatic and supportive treatments were also administered, but there was no improvement. Further investigations, including urine and blood cultures, were performed, and the patient was started on antibiotics according to sensitivity results. Although the fever showed some improvement, it persisted. Consequently, consultations with relevant departments were requested. Despite broad-spectrum antibiotics, the patient’s condition remained refractory, and the patient was transferred to the infectious disease department. Upon admission to the infectious disease department, meropenem was continued for infection management, and additional consultations were arranged. Laboratory tests, including complete blood count, indicated elevated white blood cell count, CRP, erythrocyte sedimentation rate, ferritin, and IL-6 levels. Further investigations, including HbA1c, immunoglobulin levels, and ultrasound of the inguinal and axillary lymph nodes, were recommended. The patient was temporarily placed on antibiotics and symptomatic treatment. Ultimately, the quantitative measurement of immunoglobulin G4 (IgG4) was found to be 2.441 g/L. This case met the diagnostic criteria for IgG4-RD, including organ involvement, elevated serum IgG4 levels, and histopathological evidence of IgG4 + plasma cell infiltration. After excluding other potential causes, the final diagnosis of IgG4-related disease was made.

## 4. Discussion

IgG4-RD is a chronic, fibrotic autoimmune disease for which there is currently no unified international diagnostic standard. It predominantly affects middle-aged and elderly individuals, with an unclear pathogenesis. The disease typically involves 2 or more organ systems. This case represents a typical example of renal involvement in IgG4-RD.

Current theories suggest that the pathogenesis of IgG4-RD may be related to infections and immune system dysregulation. Following an infection, the immune system is activated, leading to the activation of a large number of lymphocytes that release cytokines such as interleukin (IL)-4, IL-6, IL-10, IL-13, and transforming growth factor-β.^[8–10]^ This results in an abnormal immune response, leading to characteristic organ fibrosis and plasma cell infiltration.^[11]^ IgG4-RD is generally characterized by plasma cell infiltration in affected organs, elevated serum IgG4 levels, and chronic fibrosis, but its clinical manifestations are diverse, often leading to misdiagnosis as infections or tumors.

In this case, the patient presented with persistent high fever and a left perirenal mass, initially misdiagnosed as a perirenal abscess and treated with antibiotics and supportive care with no significant improvement. After being referred to the infectious disease department, further laboratory tests were conducted, revealing elevated levels of IL-6 (263.00 pg/mL, normal < 7 pg/mL), IL-8 (68.88 pg/mL, normal < 20.60 pg/mL), PR3-ANCA (>400.00 RU/mL, normal < 20 RU/mL), and immunoglobulin G4 (IgG4) at 2.441 g/L. Imaging findings (CT and MRI) also supported the diagnosis of IgG4-RD. This underscores the importance of considering autoimmune etiologies, especially IgG4-RD, in cases of unexplained fever.

The literature indicates that IgG4-RD can affect multiple organs, including the kidneys, lungs, and lymph nodes. In this case, the patient’s chest CT revealed bilateral pulmonary interstitial changes, multiple calcified foci in the lungs, and small nodules, with additional calcification in the liver, further supporting the multi-organ involvement characteristic of IgG4-RD.

The spectrum of renal involvement in IgG4-related disease (IgG4-RD) is broad, yet it is overwhelmingly dominated by tubulointerstitial nephritis (IgG4-TIN), which accounts for the majority of cases and is often characterized by renal dysfunction, hypocomplementemia, and diffuse parenchymal lesions on imaging. In stark contrast, our case presented as a solitary, mass-forming perirenal lesion, a phenotype that is exceedingly rare and can easily be mistaken for a malignancy or a conventional abscess. This stark difference in radiographic presentation underscores the remarkable heterogeneity of IgG4-RD and its capacity to masquerade as other common surgical or infectious conditions.

Furthermore, the serological profile in our case deviated from the classic IgG4-TIN pattern. While most patients with IgG4-TIN present with hypocomplementemia, reflecting immune complex deposition, our patient’s C3 and C4 levels were within normal limits. This suggests the absence of significant immune complex-mediated glomerular damage, which is a common feature in TIN but may be less prominent in other forms of renal IgG4-RD. Instead, the significantly elevated IgE levels observed in our patient suggest a predominant activation of a Th2-type immune response, potentially indicating an “allergic” or inflammatory subtype of IgG4-RD, as reported in the literature. This unique combination of a mass-like morphology and a distinct serological profile (normal complement + elevated IgE) highlights a rare but important clinical variant. It emphasizes that the diagnosis of IgG4-RD should be considered even in the presence of atypical features, such as a normal complement level and a tumor-like lesion, particularly when conventional therapies for infection or suspicion of malignancy prove futile.^[12]^

It is also important to note that some IgG4-RD patients may present with weakly positive ANCA, potentially linked to widespread immune system activation.^[13]^ Although this patient had positive IgM rheumatoid factor and PR3-ANCA, kidney biopsy did not show necrotizing vasculitis or crescent formation, and clinical symptoms such as nasal destruction and lung cavitation, typical of granulomatosis with polyangiitis, were absent, thus excluding the diagnosis of ANCA-associated vasculitis.

In terms of treatment, after transferring to the infectious disease department, the patient was treated with 30 mg of methylprednisolone sodium succinate daily, and oral etoricoxib (60 mg) was added to manage fever. After 3 days of treatment, the patient’s temperature normalized, and his condition significantly improved. Follow-up tests revealed a reduction in IgG4 levels to 2.112 g/L, IL-6 to 78.50 pg/mL, and a significant decrease in erythrocyte sedimentation rate, neutrophil count, procalcitonin, and ferritin, indicating effective control of the inflammatory state.

Once the patient’s condition stabilized, he was discharged and continued oral methylprednisolone therapy with a tapering regimen. Regular outpatient follow-up was scheduled to monitor serum IgG4, C3, C4, and IL-6 levels to assess disease activity and recurrence risk.

This case underscores the importance of considering IgG4-RD in the differential diagnosis when encountering middle-aged and elderly patients with unexplained fever and renal dysfunction, particularly when conventional antibiotic therapy fails. Early recognition and timely initiation of immunosuppressive treatment are critical to preventing irreversible organ damage and improving patient outcomes.

## 5. Patient perspective

The patient recalled that the initial misdiagnosis caused significant psychological distress, particularly when multiple courses of antibiotic therapy failed to alleviate his symptoms. He expressed considerable frustration and anxiety during this period of uncertainty. Following the definitive diagnosis of IgG4-related disease and the initiation of immunosuppressive treatment, his symptoms improved markedly, and his quality of life was substantially restored. The patient hopes that sharing his experience will raise clinical awareness of this condition and help others with similar presentations achieve timely diagnosis and appropriate management.

## 6. Conclusion

This case highlights that IgG4-RD may present in middle-aged and elderly patients as unexplained fever and localized mass-like lesions, which are often misdiagnosed as infectious conditions. In cases where conventional antibiotic therapy fails, especially those with evidence of multi-organ involvement and abnormal immunological indicators, IgG4-RD should be considered. Early recognition and timely initiation of immunosuppressive therapy are essential for improving patient outcomes and preventing irreversible organ damage.

In conclusion, this case highlights the diagnostic pitfalls of IgG4-RD masquerading as a surgical condition and underscores the necessity for heightened clinical vigilance. The timely shift from antimicrobial to immunosuppressive therapy was pivotal in achieving a favorable outcome, preventing potential irreversible organ damage for this patient. This narrative aligns with the overarching aim of the United Nations Sustainable Development Goal 3 (SDG 3) to “ensure healthy lives and promote well-being for all at all ages.” Improving diagnostic accuracy for complex, rare diseases is a fundamental step in reducing morbidity and achieving this global health target.

## Author contributions

**Conceptualization:** Zhongze Zhou, Fengxiang Gan, Kun Zhao, Shuai Liu, Wenxuan Li, Xinling Han, Zhilong Dong.

**Data curation:** Zhongze Zhou, Fengxiang Gan, Kun Zhao, Shuai Liu, Wenxuan Li, Xinling Han, Zhilong Dong.

**Formal analysis:** Zhongze Zhou, Xi Xiao.

**Funding acquisition:** Xi Xiao.

**Investigation:** Zhongze Zhou, Fengxiang Gan.

**Methodology:** Zhongze Zhou.

**Project administration:** Zhilong Dong.

**Resources:** Zhilong Dong.

**Software:** Zhilong Dong.

**Supervision:** Fengxiang Gan, Kun Zhao, Wenyun Wang.

**Validation:** Fengxiang Gan, Wenyun Wang.

**Visualization:** Shuai Liu, Xi Xiao, Zhilong Dong.

**Writing – original draft:** Zhongze Zhou.

**Writing – review & editing:** Zhongze Zhou, Zhilong Dong.
